# Supplementary material for: Bisnorgammacerane traces predatory pressure and the persistent rise of algal ecosystems after Snowball Earth
Source: Nat Commun. 2019 Jan 29;10:476. doi: 10.1038/s41467-019-08306-x (PMC6351664; doi:10.1038/s41467-019-08306-x)
Supplement: Supplementary file 1 — Supplementary Information [file 41467_2019_8306_MOESM1_ESM.pdf]

Supplementary Information for

Bisnorgammacerane traces predatory pressure and the persistent rise of algal ecosystems after Snowball Earth

Van Maldegem et al.

Supplementary Methods

Araras Group samples. Samples from the Araras Group were collected in 2009 from the Terconi Quarry (15º40’42”S, 58º04’32”W), located southwest of the town of Mirassol d’Oeste, Mato Grosso, Brazil. Here ~35 m of stratigraphy is exposed in outcrop, including the contact between the Marinoan glacial diamictite (Puga Formation) and pink dolomite of the Mirassol d’Oeste Formation cap carbonate[1]. The carbonates in the Terconi Quarry are among the thermally best preserved deposits of the early Ediacaran, displaying Rock Eval T_{MAX} values < 440ºC (average 417ºC)[2,3]. These values point to thermal immaturity from an oil window perspective and imply that these rocks should have preserved indigenous polycyclic terpenoid biomarkers. Sedimentological observations leave little doubt that the Mirassol d’Oeste Formation is a Snowball Earth related cap carbonate[2,4]. Values between 0.7074 and 0.7085 for the ratio of $^{87}Sr/^{86}Sr$[2] point to an early Ediacaran age of the Mirassol d’Oeste Fm[5], while Pb/Pb dates of 627 (± 32) Ma and 622 (± 33) Ma obtained on samples from the lowest Guia Fm[6], additionally confirm that this unit is a post-Marinoan cap carbonate.

Chuar Group samples. The sedimentary sequence of the Chuar Group represents mudrock interbedded with centimeter to meter thick sandstone and carbonate beds[7] and comprises one of the best-preserved Neoproterozoic records to investigate ecologies[8], climate and environmental conditions[9,10] during the break-up of Rodinia and prior to the first Neoproterozoic low latitude glaciation[11]. We collected 55 carbonate and shale samples, covering >700 m of the upper Chuar Group from the northwest flank of Nankoweap Butte (36º16’22” N, 111º53’ 29” W), Grand

Canyon, USA, in May 2014. An excellent thermal preservation is evident from Rock Eval T_{max} values below 445ºC. The age of the Chuar Group is well constrained through a detrital zircon age of 742 ± 6 Ma obtained on a tuff bed near the top of the Kwagunt Formation[12]. A shallow depositional environment that formed within an intracratonic basin in response to the break-up of Rodinia[7,13] is evident from gypsum pseudomorphs, potential paleosols and frequent desiccation features in the lower part of the Chuar Group, while microfossils[8] and cyclostratigraphic clues[14] suggest a repeated connection to open marine waters. Black shales in the Walcott member are thought to have been deposited during a marine transgression[10] and display interbedded oolite, bituminous calcite and carbonate layers. The Walcott member is capped by the Sixty Mile Formation, a sandstone and breccia interbedded succession[15].

Contamination control. Procedural blanks were worked up in parallel to the samples with two blanks employed per batch of five samples. In all cases their accumulated hydrocarbon content was significantly lower than the amount of bitumen contained in samples, thereby indicating that the laboratory workup did not add any significant or relevant contamination to the samples (Fig. S2). All samples were separated into interior and exterior portions, either by sawing or by micro-ablation, which were worked up and analysed separately. As previously outlined by Brocks and colleagues[16,17] the distribution of hydrocarbons and (if present) plastic-derived contaminants can frequently reveal if samples are partially (*i.e.* externally) or pervasively contaminated. Such comparisons showed no significant differences for Araras Group samples with high organic matter content (n=5; Fig. S2) or for any of the Chuar Group samples. A difference was observed for those samples of the Araras Group that were low in organic matter content (n=6; Fig. S2), where elevated n-alkanes and a UCM are present in the exterior. Here, the additional presence of

exclusively plastic-derived branched alkanes with quaternary carbon atoms (BAQCs) in the exterior of samples, but not in their interior, allowed us to validate the integrity of the hydrocarbon signal found in the interiors of samples[17]. Given that the Araras samples were collected as fresh and unweathered material in a quarry with recent activity, we attribute their external contamination to temporary storage in plastic bags[18]. Samples of the Chuar Group were wrapped in aluminium foil and stored in cotton bags; they never witnessed close contact to plastics. Our analysis of the Araras samples showed that the lowest four samples (< 11.0 m) did not contain any indigenous biomarkers, above 11.0 m two samples did show external contamination, yet we assess the interior organic matter to be indigenous, whereas the top five samples did not show any overprint from contaminants. For this study we have only used organic information acquired from interior material.

Structural elucidation of 25,28-bisnorgammacerane. Structural elucidation of BNG was performed using 1D and 2D microcryoprobe NMR spectroscopy (Figs. 2, S3, S4 and S5). The ^{1}H NMR spectrum showed only signals in the aliphatic region. Only 14 carbons were observed in the HSQC and HMBC spectra, suggesting a symmetrical molecule with six methyl, 12 methylene, six methine, and four quaternary carbons (*Supplementary Table 1*). Detailed analysis of COSY, HSQC, and HMBC correlations suggested a pentacyclic triterpane structure with only six-membered rings. HMBC correlations indicated that the methyl groups are located in positions 4 (C-23 and C-24), 8 (C-26), 14 (C-27) and 22 (C29 and C30), establishing the constitution of the molecule as 25,28-bisnorgammacerane (Figs. 2, S3, S4 and S5).

This constitution is only compatible with C_2 symmetry. The conformation (trans-fused chairs) and relative configuration could be established by the observation of large (> 10 Hz) axial/axial $^3J_{HH}$ couplings for H_α-3, H_β-2, H_α-1, H_β-10, H_α-5, H_β-6, H_α-7, H_α-9, and H_β-11. The relative configuration was further corroborated by determination of the spatial proximity of protons. NOESY correlations were observed between the axial 24-methyl group and H-10 and between H-10 and the axial 26-methyl group as well as between H-5 and H-9. However, no NOESY correlation was observed between the 24-methyl group and H-5, indicating that 24- and 26-methyl groups and H-10 are at the same side and H-5 and H-9 are at the opposite side of the molecule (Fig. 2). In addition, a strong NOE was observed between the two equatorial protons H_β-1 and H_α-11. Further support for the conformation and configuration came from the HMBC correlations of methylene protons, showing distinct $^3J_{CH}$ correlations for equatorial protons and $^2J_{CH}$ correlations for axial protons (*Supplementary Table 1*). Thus, the configuration of BNG was determined to be either:

5α(H),8β,9α(H),10β(H),13β(H),14α,17β(H),18α(H) or

5β(H),8α,9β(H),10α(H),13α(H),14β,17α(H),18β(H).

Since no isomers of BNG were observed, it is very likely that the primary configuration of the BNG precursors tetrahymanol and gammacerane[19] is preserved and the configuration of BNG is 5α(H),8β,9α(H),10β(H),13β(H),14α,17β(H),18α(H).

Supplementary Note 1: Self-sustaining cyanobacterial feedback loop

Prior to the Late Neoproterozoic rise of eukaryotic algae to ecological significance[20], primary productivity was likely dominated by cyanobacteria[20–24], which are expected to ecologically outcompete eukaryotic algae under the nutrient poor conditions that existed before the Cryogenian[25]. Under this regime, Earth's oceans likely witnessed a closed positive feedback loop of self-sustained bacterial dominance. For one, dense communities of pelagic cyanobacteria preclude photosynthetic eukaryotic algae from becoming relevant primary producers by significant shading, given that algae have significantly higher light requirements[23]. Cyanobacteria predominantly generate dissolved organic matter (DOM) and slow sinking particulate organic matter (POM), which would have led to increased consumption of water-column oxygen in surface waters[22] and potentially to widespread oceanic stratification[26]. Low net carbon export rates and consequent burial would have prevented atmospheric oxygenation and in this situation, essential nutrients such as phosphorus would have remained scarce[20–22,25]: under the ensuing anoxic ferruginous conditions, which were widespread during the Proterozoic[10,25], phosphorus would have been efficiently scavenged as ferrous phosphates or by co-precipitation with iron oxides[25]. It was suggested that massive glaciogenic phosphorus input during the Snowball Earth deglaciation phases would have tipped this system in favour of eukaryotes[20]. With the rise of algae a new transportation mechanism emerged, allowing the larger particulate organic matter (POM) to sink faster through the water column providing bioavailable nutrients to the bottom waters, breaking up the cyanobacterial feedback loop, and providing surface water oxygenation. But in contrast to the phosphorus record[25], which places nutrient regime change into the Cryogenian, the biomarker record does not indicate a significant

shift in primary producers during this era. Besides one sample from the Ghadir Manqil Fm., Oman[27], sedimentary abundances of eukaryote-derived steranes remain low during the Cryogenian interglacial, suggesting that the majority of ecosystems remained cyanobacterial-dominated until after the demise of the second of the Snowball Earth glaciations. Other studies have suggested that the self-sustaining cyanobacterial loop can only be broken by biology: the action of filter feeding animals (*i.e.* in the form of the earliest sponges)[23], or predatory protists[22] would have capped cyanobacterial population sized sufficiently for eukaryotic algae to rise to ecological dominance.

Supplementary Note 2: Stable carbon isotope systematics ($\Delta\delta^{13}C_{A\text{-}K}$)

A change in the intensity of the Proterozoic biological pump was suggested to be recorded in the relationship between bulk organic carbon isotopes ($\delta^{13}C_{Ker}$) and the compound specific isotope values of *n*-alkanes ($\delta^{13}C_{Alk}$)[26]. Bulk organic matter (*i.e.* kerogen) represents an amalgam, mainly derived from primary produced biomass and is formed during the early stages of diagenesis[28,29], whereas *n*-alkanes largely derive from fatty acids that are produced by both primary producing autotrophs and by heterotrophs. Autotrophs, which use the Calvin-Benson-Bassham cycle to sequester carbon (*e.g.* cyanobacteria among others), will typically generate fatty acids ~4‰ lighter compared to the bulk organic matter[30–32]. Heterotrophic organisms, in contrast, sequester carbon by feeding on the carbon available in the DOM or POM pool. During the metabolism of heterotrophs isotopically lighter CO_2 is excreted, resulting in an overall $\delta^{13}C$ enrichment in their lipids of ~1–2 ‰ relative to the initial carbon source[33]. As mentioned above, the biological

community in the early Proterozoic largely consisted of small bacterial organisms, limiting the strength of the biological pump and allowing heterotrophic organisms to consistently feed on the available POM and DOM pools, resulting in a continuous contribution of isotopically enriched heterotrophic derived lipids and fatty acids into the organic matter pool. Logan and colleagues[26] observed that throughout the early and mid-Proterozoic *n*-alkanes displayed a significant isotopic enrichment relative to kerogen, which disappeared during the late Neoproterozoic[26]. This shift in the relative offset between kerogen and compound specific *n*-alkanes stable carbon isotope values (expressed as $\Delta\delta^{13}C_{A\text{-}K}$) seen at the end of the Proterozoic was attributed to the evolution of a digestive system and fecal pellets, allowing organic matter to sink much faster through the water column. As a consequence, lower exposure to potential heterotrophic recycling led to an overall reduction in the relative enrichment of the lipids and fatty acids[26]. However, concerns have been raised about this hypothesis as it would require the entire Proterozoic to have witnessed the unlikely circumstance of highly stable diagenetic conditions for over a billion years[34]. Instead, an alternative theory explaining the shift in $\Delta\delta^{13}C_{A\text{-}K}$ was brought forward by Close and colleagues[34]. Using a modelling approach they pointed out that organic matter derived from a mixed community (*i.e.* bacteria and eukaryotic organisms) provides a substantially smaller $\Delta\delta^{13}C_{A\text{-}K}$ offset compared to organic matter derived from a bacterially dominated system, implying that a shift in $\Delta\delta^{13}C_{A\text{-}K}$ is more plausibly reflective of a change in the community composition[34]. In our study we observe a significant change in $\Delta\delta^{13}C_{A\text{-}K}$ (Fig. 4), which seemingly co-varies with changes in both heterotrophic cycling and community composition suggesting that both mechanisms could have influenced the observed isotopic signatures.

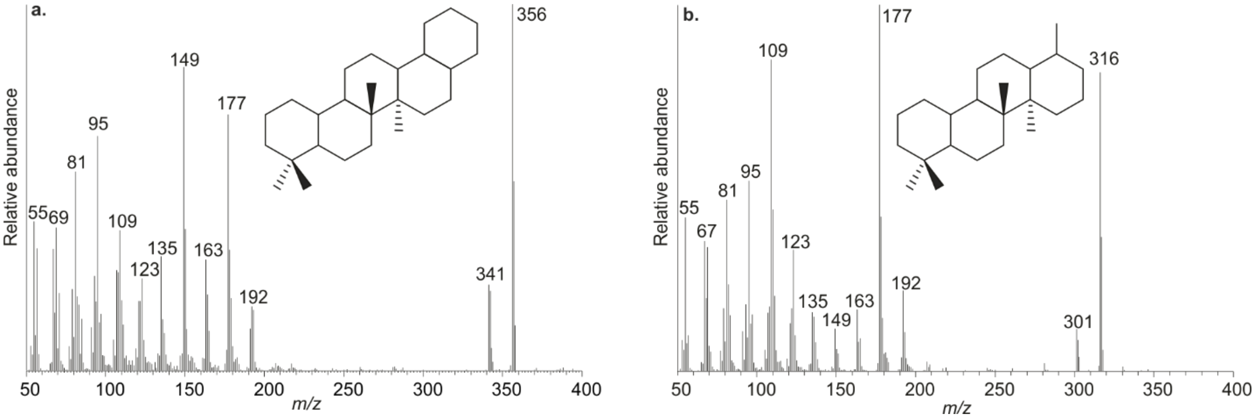

Supplementary Figure 1: Two related polycyclic terpenoids likely represent further degradation products of 25,28-bisnorgammaerane (a.) mass spectra (electron impact: 70 eV) and possible structure of a C_{26} polycyclic molecule, which we tentatively identified as tetranorgammacerane; (b.) mass spectra (electron impact: 70 eV) and possible structure of a tetracyclic C_{23} compound that we tentatively identified as 25-nor-des-E-gammacerane.

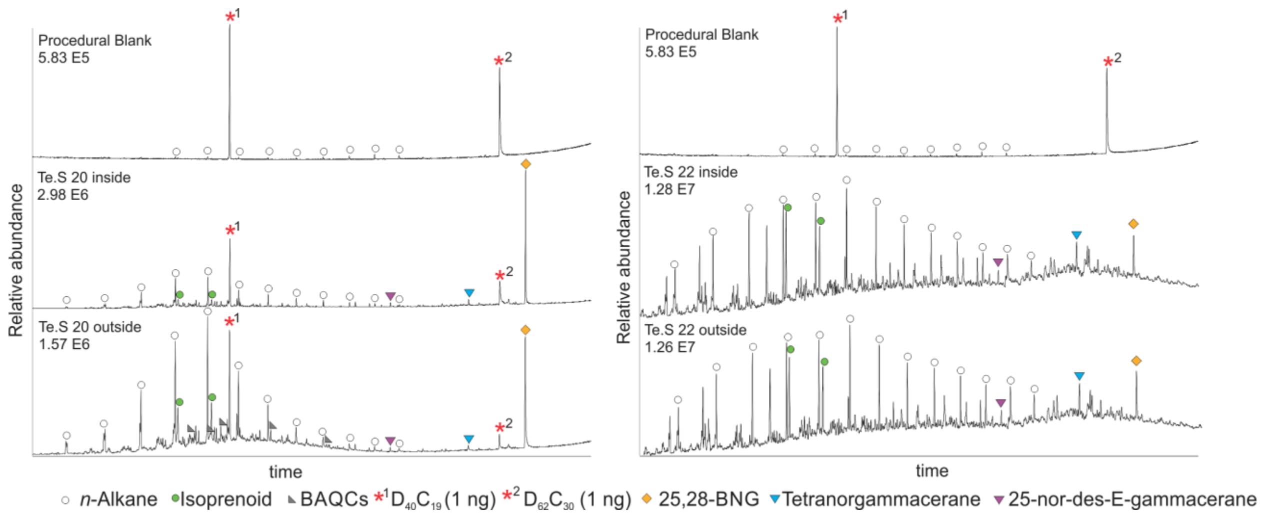

Supplementary Figure 2: Sample interiors *vs.* exteriors *vs.* procedural blanks. Total ion GC-MS chromatograms (TIC; *m/z* 50–550) comparing the interior and exterior rock extracts, and a procedural blank of two samples from the Mirassol d'Oeste Formation. Sample Te.S 20 (11.7 m, left side) yields relatively little hydrocarbons and displays overprint by BAQCs (grey triangles) only on the outside, whereas sample Te.S 22 (12.7 m, right side) is relatively rich and does not display any traceable external contamination (see further *Supplementary Methods*). Alongside BNG commonly two additional, unusual polycyclic terpanes are detected which, based on their mass spectra (Fig. S1), are tentatively identified as tetranorgammacerane ($C_{26}H_{44}$; *m/z* 356; blue triangles) and 25-nor-des-E-gammacerane ($C_{23}H_{40}$; *m/z* 316; purple triangles).

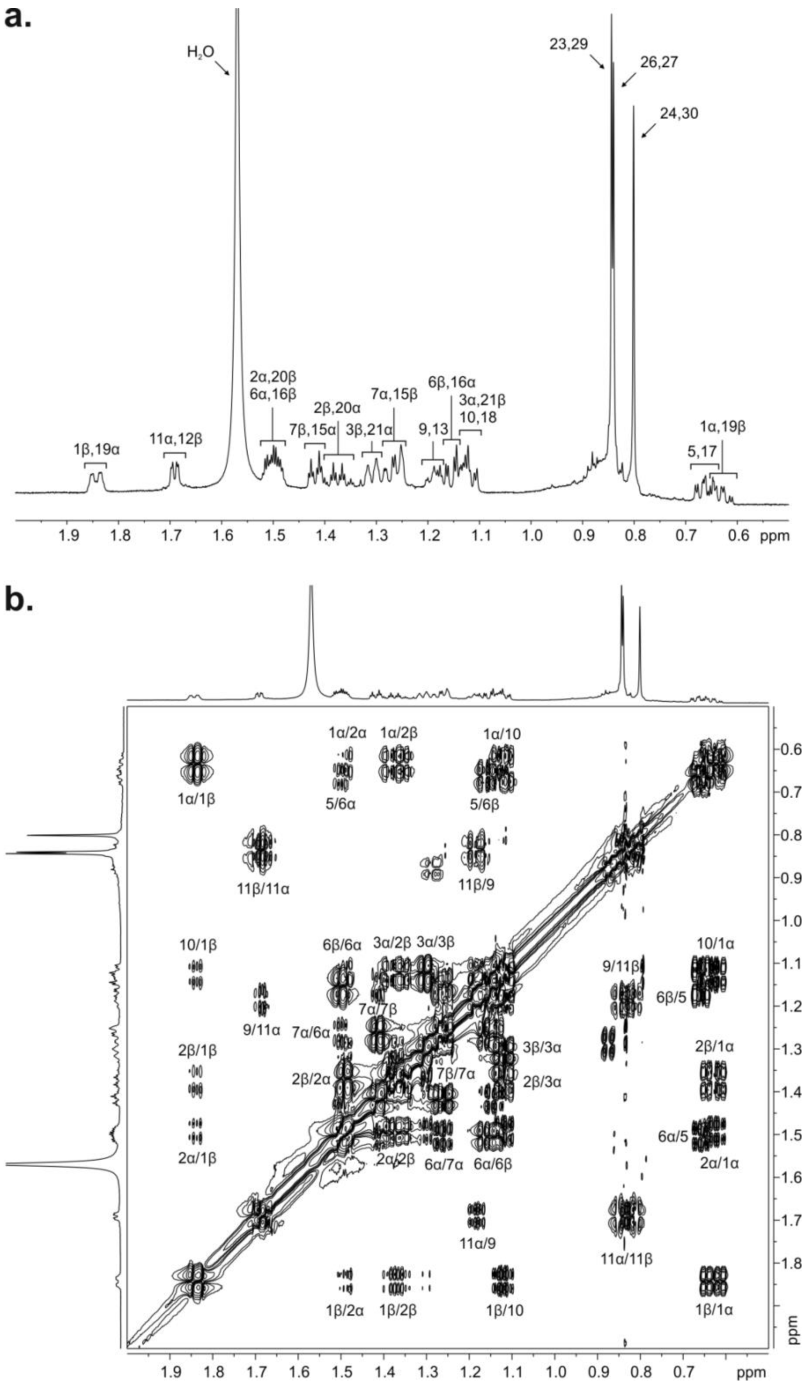

Supplementary Figure 3: NMR spectra of BNG. (a) ^{1}H NMR spectrum ($CDCl_3$, 800 MHz); (b) ^{1}H-^{1}H COSY spectrum ($CDCl_3$, 800 MHz) (For assignment and structural elucidation see Supplementary Information and Supplementary Table 1).

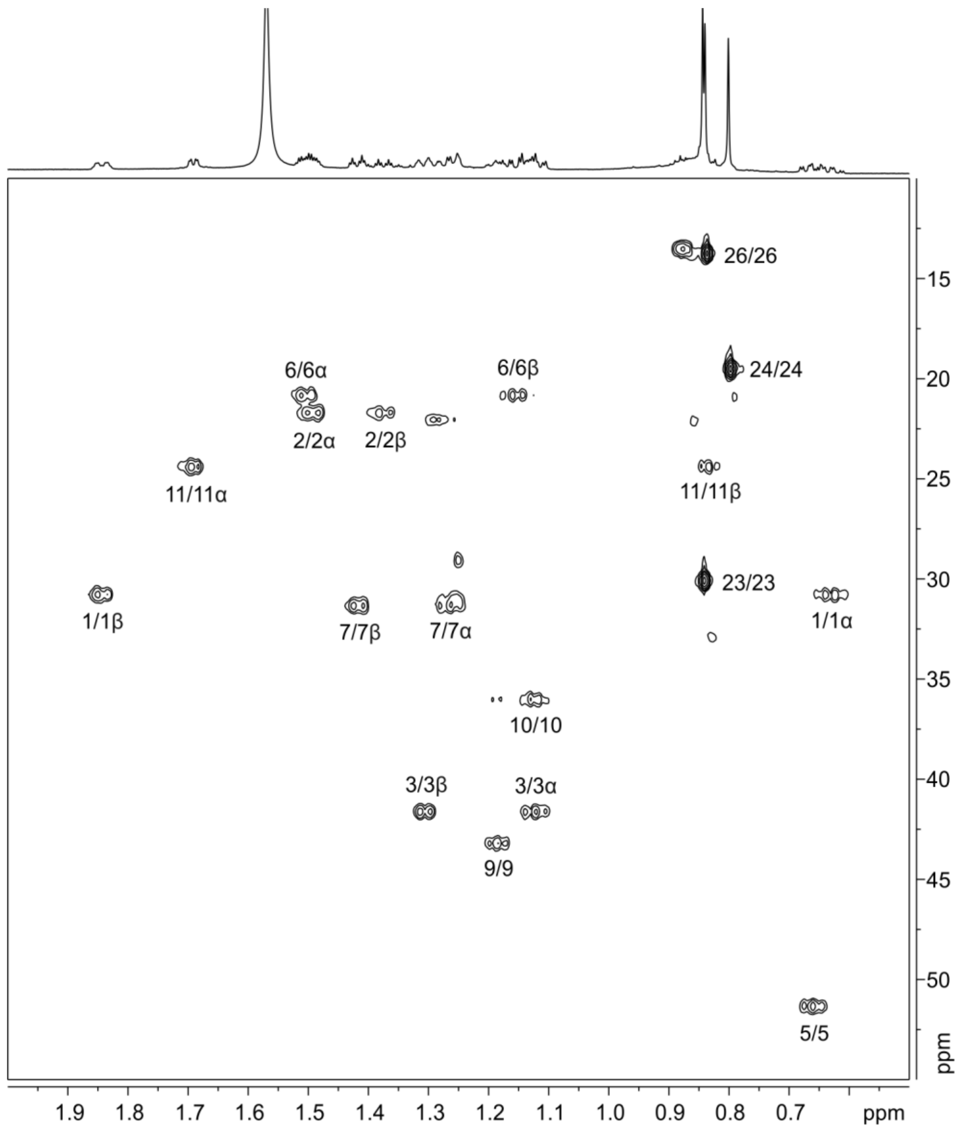

Supplementary Figure 4: HSQC spectrum. ^{1}H-^{13}C HSQC spectrum ($CDCl_3$, 800 MHz) of 25,28-bisnorgammacerane (For assignment and structural elucidation see Supplementary Information and Supplementary Table 1).

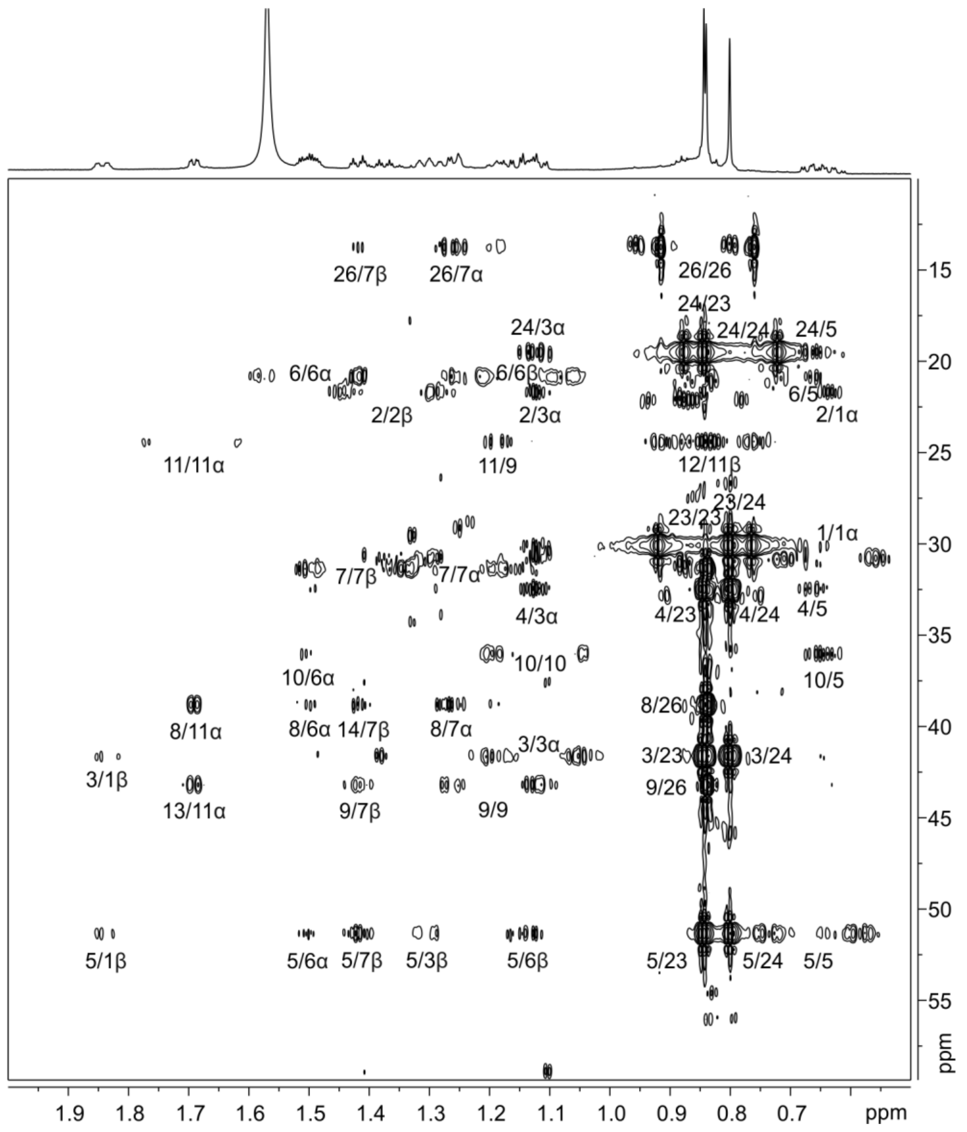

Supplementary Figure 5: HMBC spectrum. ^{1}H-^{13}C HMBC NMR spectrum ($CDCl_3$, 800 MHz) of 25,28-bisnorgammacerane (For assignment and structural elucidation see Supplementary Information and Supplementary Table 1).

Supplementary Table 1: NMR spectroscopic data for 25,28-bisnorgammacerane ($CDCl_3$, 800 MHz). All chemical shifts were referenced to $CHCl_3$ (δ_H = 7.26 ppm, δ_C = 77.16 ppm).

Position	δ_C[a]	δ_H (mult., J in Hz)	HMBC[b]
1	30.8, CH_2	α, ax. 0.63[c]	2,10
		β, eq. 1.84	3, 5
2	21.7, CH_2	α, eq. 1.49[c]	4
		β, ax. 1.37 (qt, 13.6, 3.7)	1, 3
3	41.6, CH_2	α, ax. 1.13[c]	2, 4, 24
		β, eq. 1.31	5, 1
4	32.5, qC	–	–
5	51.3, CH	α, ax. 0.66[c]	4, 6, 10, 24
6	20.8, CH_2	α, eq. 1.50[c]	5, 8, 10
		β, ax. 1,15[c]	5
7	31.3, CH_2	α, ax. 1.27[c]	8, 26
		β, eq. 1.42 (dt, 12.4, 3.4)	5, 9, 14, 26
8	38.8, qC	–	–
9	43.2, CH	α, ax. 1.19[c]	11
10	36.0, CH	β, ax. 1.13[c]	
11	24.4, CH_2	α, eq. 1.69 (dd, 8.7, 2.6)	8, 13
		β, ax. 0.84[c]	12
12	24.4, CH_2	α, ax. 0.84[c]	11
		β, eq. 1.69 (dd, 8.7, 2.6)	9, 14
13	43.2, CH	β, ax. 1.19[c]	12
14	38.8, qC	–	–
15	31.3, CH_2	α, eq. 1.42 (dt, 12.4, 3.4)	8, 13, 17, 27
		β, ax. 1.27[c]	14, 27
16	20.8, CH_2	α, ax. 1,15[c]	17
		β, eq. 1.50[c]	14, 17, 18
17	51.3, CH	β, ax. 0.66[c]	16, 18, 22, 30
18	36.0, CH	α, ax. 1.13[c]	
19	30.8, CH_2	α, eq. 1.84	17, 21
		β, ax. 0.63[c]	18, 20
20	21.7, CH_2	α, ax. 1.37 (qt, 13.6, 3.7)	19, 21
		β, eq. 1.49[c]	22
21	41.6, CH_2	α, eq. 1.31	17, 19
		β, ax. 1.13[c]	20, 22, 30
22	32.5, qC	–	–
23	30.1, CH_3	α, eq. 0.844 (s)	3, 4, 5, 24
24	19.5, CH_3	β, ax. 0.800 (s)	3, 4, 5, 23
26	13.8, CH_3	β, ax. 0.840 (s)	7, 8, 9
27	13.8, CH_3	α, ax. 0.840 (s)	13, 14, 15
29	30.1, CH_3	β, eq. 0.844 (s)	17, 21, 22, 30
30	19.5, CH_3	α, ax. 0.800 (s)	17, 21, 22, 29

[a]δ_C values determined from HSQC and HMBC spectra.

[b]HMBC correlations are from proton(s) stated to the indicated carbon.

[c]Overlapped signals in ^1H NMR spectrum, δ_H values determined from HSQC spectrum.

Supplementary References:

1. Sansjofre, P. *et al.* Paleoenvironmental reconstruction of the Ediacaran Araras platform (Western Brazil) from the sedimentary and trace metals record. *Precambrian Res.* **241,** 185–202 (2014).

2. Sansjofre, P. *et al.* A carbon isotope challenge to the snowball Earth. *Nature* **478,** 93–96 (2011).

3. Sousa Júnior, G. R. *et al.* Organic matter in the Neoproterozoic cap carbonate from the Amazonian Craton, Brazil. *J. South Am. Earth Sci.* **72,** 7–24 (2016).

4. Nogueira, A. C. R. *et al.* Carbon and strontium isotope fluctuations and paleoceanographic changes in the late Neoproterozoic Araras carbonate platform, southern Amazon craton, Brazil. *Chem. Geol.* **237,** 186–208 (2007).

5. Halverson, G. P. & Shields-Zhou, G. Chemostratigraphy and the Neoproterozoic glaciations. *Geol. Soc. London, Mem.* **36,** 51–66 (2011).

6. Babinski, M. *et al.* Chronology of Neoproterozoic ice ages in central Brazil. *SSAGI, VI South Am. Symp. Isot. Geol.* (2006).

7. Dehler, C. M. *et al.* Neoproterozoic Chuar Group (~800-742 Ma), Grand Canyon: A record of cyclic marine deposition during global cooling and supercontinent rifting. *Sediment. Geol.* **141–142,** 465–499 (2001).

8. Porter, S. M. Tiny vampires in ancient seas: evidence for predation via perforation in fossils from the 780–740 million-year-old Chuar Group, Grand Canyon, USA. *Proc. R. Soc. B Biol. Sci.* **283,** 1–6 (2016).

9. Nagy, R. M., Porter, S. M., Dehler, C. M. & Shen, Y. Biotic turnover driven by eutrophication before the Sturtian low-latitude glaciation. *Nat. Geosci.* **2,** 415–418 (2009).

10. Johnston, D. T. *et al.* An emerging picture of Neoproterozoic ocean chemistry: Insights from the Chuar Group, Grand Canyon, USA. *Earth Planet. Sci. Lett.* **290,** 64–73 (2010).

11. Hoffman, P. F., Kaufman, A. J., Halverson, G. P. & Schrag, D. P. A Neoproterozoic Snowball Earth. *Science (80-.).* **281,** 1342–1346 (1998).

12. Karlstrom, K. E. *et al.* Chuar Group of the Grand Canyon: Record of breakup of Rodinia, associated change in the global carbon cycle, and ecosystem expansion by 740 Ma. *Geology* **28,** 619–622 (2000).

13. Timmons, J. M., Karlstrom, K. E., Dehler, C. M., Geissman, J. W. & Heizler, M. T. Proterozoic multistage (ca. 1.1 and 0.8 Ga) extension recorded in the Grand Canyon Supergroup and establishment of northwest- and north-trending tectonic grains in the southwestern United States. *Bull. Geol. Soc. Am.* **113,** 163–180 (2001).

14. Dehler, C. M. *et al.* High-resolution δ13C stratigraphy of the Chuar Group (ca. 770-742 Ma), Grand Canyon: Implications for mid-Neoproterozoic climate change. *Bull. Geol.

Soc. Am. **117,** 32–45 (2005).

15. Ford, T. D. & Breed, W. J. Late Precambrian Chuar Group, Grand Canyon, Arizona. *Geol. Soc. Am. Bull.* **84,** 1243–1260 (1973).

16. Brocks, J. J. Millimeter-scale concentration gradients of hydrocarbons in Archean shales: Live-oil escape or fingerprint of contamination? *Geochim. Cosmochim. Acta* **75,** 3196–3213 (2011).

17. Brocks, J. J., Grosjean, E. & Logan, G. A. Assessing biomarker syngeneity using branched alkanes with quaternary carbon (BAQCs) and other plastic contaminants. *Geochim. Cosmochim. Acta* **72,** 871–888 (2008).

18. Leider, A., Schumacher, T. C. & Hallmann, C. Enhanced procedural blank control for organic geochemical studies of critical sample material. *Geobiology* **14,** 469–482 (2016).

19. Hills, I. R., Whitehead, E. C., Anders, D. E., Cummins, J. J. & Robinson, W. E. An optically active triterpane gammacerane in GRS bitumen. *Chem. Commun.* 752–754 (1966).

20. Brocks, J. J. *et al.* The rise of algae in Cryogenian oceans and the emergence of animals. *Nature* **548,** 578–581 (2017).

21. Brocks, J. J. The transition from a cyanobacterial to algal world and the emergence of animals. *Emerg. Top. Life Sci.* 1–10 (2018).

22. Lenton, T. M. & Daines, S. J. The effects of marine eukaryote evolution on phosphorus, carbon and oxygen cycling across the Proterozoic–Phanerozoic transition. *Emerg. Top. Life Sci.* **0,** (2018).

23. Butterfield, N. J. Animals and the invention of the Phanerozoic Earth system. *Trends Ecol. Evol.* **26,** 81–87 (2011).

24. Knoll, A. H. Paleobiological perspectives on early eukaryotic evolution. *Cold Spring Harb. Perspect. Biol.* **6,** (2014).

25. Reinhard, C. T. *et al.* Evolution of the global phosphorus cycle. *Nature* **541,** 386–389 (2017).

26. Logan, G., Hayes, J. M., Hieshima, G. B. & Summons, R. E. Terminal Proterozoic reorganization of biogeochemical cycles. *Nature* **376,** 53–56 (1995).

27. Love, G. D. *et al.* Fossil steroids record the appearance of Demospongiae during the Cryogenian period. *Nature* **457,** 718–21 (2009).

28. Tegelaar, E. W., De Leeuw, J. W., Derenne, S. & Largeau, C. A reappraisal of kerogen formation. *Geochim. Cosmochim. Acta* **53,** 3103–3106 (1989).

29. De Leeuw, J. W. De *et al.* Resistant biomacromolecules as major contributors to kerogen. *Philos. Trans. R. Soc.* **333,** 329–337 (1991).

30. Hayes, J. M. Factors controlling 13C contents of sedimentary organic compounds: Principles and evidence. *Mar. Geol.* **113,** 111–125 (1993).

31. Bidigare, R. *et al.* Consisten fractionation of 13C in nature and in the laboratory: Growth-rate effects in some haptophyte algae. *Global Biogeochem. Cycles* **11,** 279–292 (1997).

32. Schouten, S. *et al.* Biosynthetic effects on the stable carbon isotopic compositions of algal lipids: implications for deciphering the carbon isotopic biomarker record. *Geochim. Cosmochim. Acta* **62,** 1397–1406 (1998).

33. DeNiro, M. J. & Epstein, S. Influence of diet on the distribution of carbon isotopes in animals. *Geochim. Cosmochim. Acta* **42,** 495–506 (1978).

34. Close, H. G., Bovee, R. & Pearson, A. Inverse carbon isotope patterns of lipids and kerogen record heterogeneous primary biomass. *Geobiology* **9,** 250–265 (2011).
