# Supplementary material for: Bisnorgammacerane traces predatory pressure and the persistent rise of algal ecosystems after Snowball Earth
Source: Nat Commun. 2019 Jan 29;10:476. doi: 10.1038/s41467-019-08306-x (PMC6351664; doi:10.1038/s41467-019-08306-x)
Supplement: Supplementary file 3 — Description of Additional Supplementary Files [file 41467_2019_8306_MOESM3_ESM.pdf]

Description of Additional Supplementary Files

File Name: Supplementary Data 1
Description: Samples investigated to determine the temporal, environmental and geographic distribution of BNG throughout the past ca. 800 Myr.
